# Cost-effectiveness analysis of anlotinib plus chemotherapy with or without benmelstobart versus chemotherapy alone for extensive-stage small-cell lung cancer in China

**DOI:** 10.3389/fonc.2024.1484650

**Published:** 2024-12-17

**Authors:** Caicong You, Jiahao Zhang, Jianying Lei, Wu Fu, Bin Zheng, Maobai Liu, Na Li

**Affiliations:** ^1^ Fujian Medical University Union Hospital, Fuzhou, Fujian, China; ^2^ The School of Pharmacy, Fujian Medical University, Fuzhou, Fujian, China

**Keywords:** cost-effectiveness, benmelstobart, anlotinib, extensive-stage small-cell lung, scenario analysis, R software, time-varying Markov

## Abstract

**Objectives:**

The ETER701 trial demonstrated that benmelstobart combined with anlotinib and etoposide-carboplatin (EC) significantly extends survival in patients with extensive-stage small cell lung cancer (ES-SCLC), setting a new record for median overall survival. In contrast, anlotinib plus EC only significantly prolongs progression-free survival. However, there is currently no evidence evaluating the cost-effectiveness of these regimens as first-line treatments. Therefore, this study assesses the cost-effectiveness of these three first-line treatment options from the perspective of the Chinese healthcare system.

**Methods:**

A time-varying Markov model was constructed to simulate the disease progression of a 62-year-old patient with ES-SCLC, assessing direct medical costs, health benefits, and incremental cost-effectiveness ratios (ICER). Both flexible and standard parametric models were included to fit and extrapolate survival data. The probabilities, costs, and health utilities required for the model were sourced from literature, databases, and expert consultations. Additionally, sensitivity and scenario analyses were conducted to explore the impact of various parameters on model uncertainty.

**Results:**

Compared to EC alone, the combination of benmelstobart, anlotinib, and EC added $80,879.12 in cost for 0.7288 quality-adjusted life years (QALYs), an ICER of $110,970.19/QALY. Anlotinib plus EC added $4,107.86for 0.1951 QALYs, an ICER of $21,056.19/QALY. At a $37,598/QALY threshold, the cost-effectiveness probability for benmelstobart combination is 0%, and for anlotinib combination is 80.42%. A 73.79% price cut for benmelstobart is needed for cost-effectiveness.

**Conclusions:**

In China, benmelstobart combined with anlotinib and EC is not a cost-effective first-line treatment for ES-SCLC; however, reducing the price of benmelstobart by 73.79% could make this regimen cost-effective. In contrast, anlotinib combined with EC may represent a more cost-effective first-line treatment option.

## 1 Introduction

Lung cancer is the most common cancer in China and the leading cause of cancer death among both men and women. In 2022, there were 1,060,600 new cases and 733,300 deaths (1). Small Cell Lung Cancer (SCLC) is a highly aggressive malignant tumor, accounting for approximately 15% of all lung cancers, with a very poor prognosis (2). About two-thirds of SCLC patients are diagnosed with extensive-stage SCLC (ES-SCLC), and the 5-year overall survival (OS) rate is limited to only 1-5% (3).

Currently, both Chinese and American clinical guidelines recommend durvalumab or atezolizumab combined with an etoposide-carboplatin regimen (EC) as the first-line treatment for ES-SCLC (4, 5). The ETER701 trial (ClinicalTrials.gov Identifier: NCT04234607) compared the efficacy of benmelstobart plus anlotinib plus EC, anlotinib plus EC, and EC alone as first-line treatments for ES-SCLC patients. The study demonstrated that the multitarget anti-angiogenic agent anlotinib, in combination with immune checkpoint inhibitors (ICIs), has a synergistic effect, enhancing the efficacy of the ICI and EC combination therapy (6). Compared to EC alone, the benmelstobart plus anlotinib plus EC regimen extended median OS (19.3 months vs. 11.9 months; hazard ratio [HR] 0.61 [0.47-0.79]) and median progression-free survival (PFS) (6.9 months vs. 5.6 months; HR 0.32 [0.26-0.41]). The anlotinib plus EC regimen also extended median OS (13.3 months vs. 11.9 months; HR 0.86 [0.67-1.10]) and median PFS (5.6 months vs. 4.2 months; HR 0.44 [0.36-0.55]) (6).

Although the benmelstobart plus anlotinib plus EC regimen achieved a higher median OS compared to previous randomized trials, benmelstobart was only approved by the Chinese National Medical Products Administration (NMPA) for the first-line treatment of ES-SCLC in May 2024. Its cost-effectiveness remains unclear. Furthermore, while anlotinib alone is the preferred third-line treatment for ES-SCLC (4), its cost-effectiveness in combination with EC as a first-line treatment has not been evaluated. Therefore, this study aims to assess the cost-effectiveness of these three treatment regimens for first-line treatment of ES-SCLC from the perspective of the Chinese healthcare system.

## 2 Materials and methods

### 2.1 Model and patient queue

**Figure 1:**
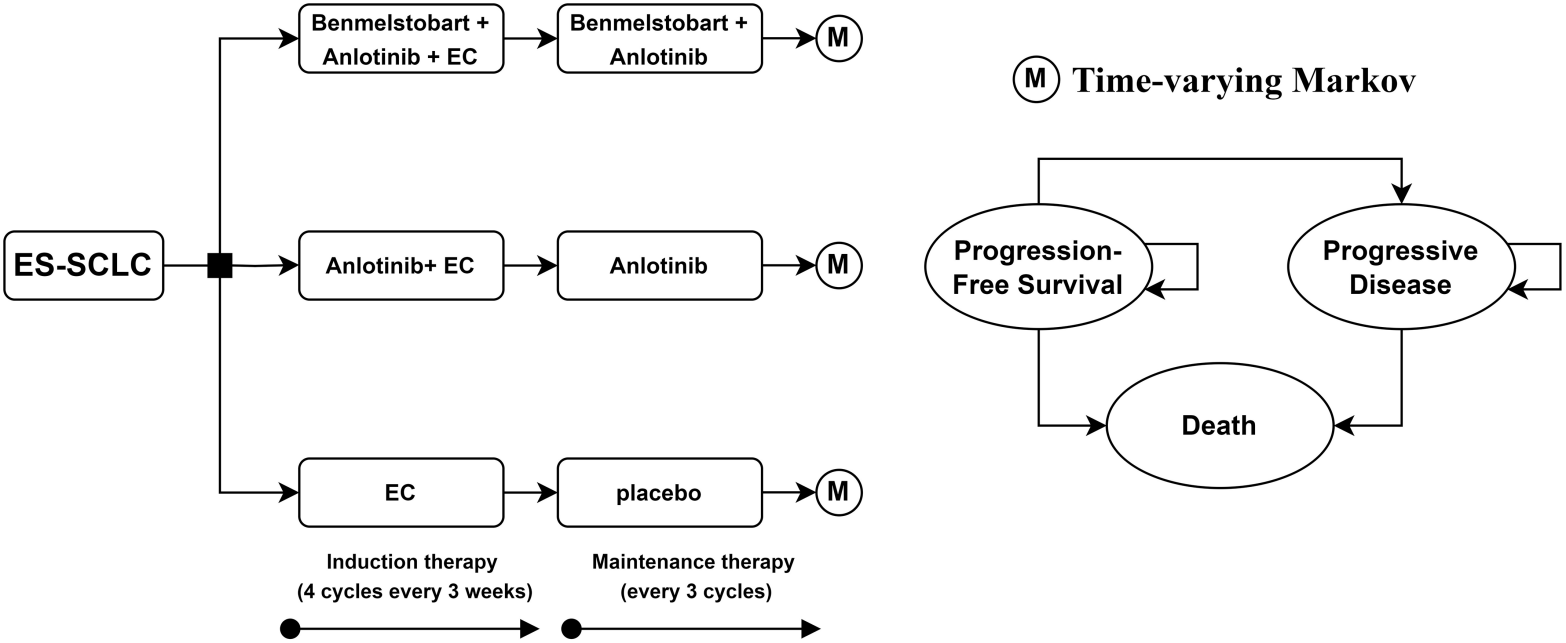
Decision tree and time-varying Markov model plots for the use of potential treatment pathways in patients with extended-stage small cell lung cancer. ES-SCLC, extensive-stage small cell lung cancer; EC, etoposide-carboplatin.

This study adheres to the 2022 Consolidated Health Economic Evaluation Reporting Standards (CHEERS 2022) health economic evaluation guidelines (**Supplementary Table 1** in the **Supplementary Information**) (7). Time-varying Markov models were constructed using the “heemod” package in R (R package version 1.0.1.9), employing the life table method to calculate state membership (8, 9). A cohort of ES-SCLC patients with a median age of 62 was established. These patients had not received any systemic treatment for ES-SCLC previously and had at least a 6-month treatment-free interval from their last therapy to their diagnosis. After diagnosis, patients were assigned to one of three maintenance treatment regimens: bendamustine plus anlotinib and EC, anlotinib plus EC, or EC alone. This study evaluates the cost-effectiveness of these three treatment strategies from the perspective of the Chinese healthcare system. The model code is provided in **Supplementary Method 1** of **Supplementary Information**. For ease of cost calculation, the model cycle length was set to 21 days. According to the ETER701 trial, the dosage per cycle for benmelstobart, anlotinib, etoposide, and carboplatin was 1200 mg on day 1, 12 mg on days 1-14, 100 mg/m2 on days 1-3, and AUC 5 mg/mL*min on day 1, respectively (6). As shown in **Figure 1**, the EC regimen was administered for four cycles, while benmelstobart and anlotinib were continued until disease progression.

Patients’ states in the model were simplified into three health states: PFS, progressive disease (PD) and death. Given that the 5-year overall survival rate for ES-SCLC patients is only 1-5% (3), the model’s time horizon was set to 10 years, by which time the majority of patients would have died. The model details are shown in **Figure 1**.

### 2.2 Transition probability estimation

The transition probabilities were estimated based on the ETER701 trial. Due to the unavailability of precise baseline patient data from the trial, we used GetData Graph Digitizer (version 2.26, http://www.getdata-graph-digitizer.com/) to digitize the Kaplan-Meier (KM) curves. Then, we used R software (version 4.4.1, https://www.r-project.org/) to reconstruct pseudo-individual patient data (IPD) and extrapolate the KM curves.

The parametric models used included seven standard parametric models (exponential, weibull, gompertz, log-normal, log-logistic, gamma, and generalized gamma distributions) and five flexible models (fractional polynomials [FP] model, restricted cubic splines [RCS] model, Royston-Parmar [RP] model, generalized additive models [GAM], and mixture cure models [MCM]). For FP models, the best models for both first-order and second-order were included (FP1 and FP2). For RP models, the best models across all three scales (‘odds’, ‘normal’, and ‘hazard’) were carefully considered. The parametric models were selected based on a combination of the Akaike Information Criterion (AIC) and the Bayesian Information Criterion (BIC), as well as visual inspection to judge the reasonableness of survival curve fitting and extrapolation. Ultimately, the OS and PFS distributions for the benmelstobart plus anlotinib plus EC group were modeled using the RP-normal and RP-hazard distributions, respectively; the OS and PFS distributions for the anlotinib plus EC group were modeled using the RP-hazard and log-logistic distributions, respectively; and the OS and PFS distributions for the EC group were modeled using the RP-odds and RP-hazard distributions, respectively. The detailed parameters of each model and the fitting and extrapolation of KM curves for each group are shown in **Supplementary Table 2** and **Supplementary Figure 1** of **Supplementary Information**. We incorporated China’s 2022 natural mortality rate and calculated the time-varying transition probabilities between states based on the extrapolated survival data for each group (10).

### 2.3 Cost estimation

This study aimed to include all direct medical costs wherever possible. The costs encompassed treatment drugs, subsequent treatments for PD, management of adverse events, best supportive care, follow-up (including laboratory tests and imaging), and palliative care, with costs converted to US dollars at an exchange rate of 7.13 RMB per USD (as of July 1, 2024). It was assumed that the average patient had a body weight of 65 kg, a body surface area of 1.72 m², and a serum creatinine level of 88.40 μmol/L for drug dosage calculations (11). The costs of each drug were sourced from Yaozhi.com, using the median bid prices from various provinces since 2023 (12).

The frequency of drug use and subsequent treatment regimens were derived from the ETER701 trial, with best supportive care assumed for those who did not receive further treatment (6, 11). Since the ETER701 trial provided only treatment categories without specifying particular drugs, we used the recommended drugs for each category from clinical guidelines to represent subsequent treatments. Conventional radiation was used for radiotherapy, topotecan for chemotherapy, anlotinib for targeted therapy, and pembrolizumab for immunotherapy (4, 12, 13). Except for immunotherapy (pembrolizumab), which was assigned a maximum duration of use of 24 months according to clinical guidelines, other treatment regimens were assumed to continue throughout the PD phase (4). If immunotherapy was administered for more than 24 months, patients received best supportive care.

The management costs for adverse events included only grade 3 or higher events with an incidence rate of ≥ 5% (10, 11, 14, 15). Assuming that all adverse events could occur in the first cycle and persist for one cycle, the cost of each adverse event is then adjusted based on the incidence rates reported in the ETER701 trial (4). The costs for palliative care were sourced from a retrospective study on treatment costs for terminal cancer patients in China. The average palliative care cost was calculated by weighting the costs for urban and rural populations (25% and 75%, respectively) and assuming that palliative care was provided during the last three months of life (16). The follow-up frequency was set according to clinical guidelines (first year: once every 2 months; years 2-3: once every 3 months; years 4-5: once every 6 months; beyond 5 years: once annually) (4). Additionally, based on the primary recommendations in the clinical guidelines, follow-up costs were assumed to include laboratory and imaging test costs, with specific costs derived from an economic burden study on advanced lung cancer patients in China (17). Relevant parameters are detailed in **Table 1**.

**Table 1:** Model parameter inputs.

Parameter	Base case	Range		Distribution	Reference
		Low	High		
Treatment cost per cycle ($)					
Benmelstobart	3444.60	2583.45	4305.75	gamma	(11)
Anlotinib	403.46	272.08	556.66	gamma	(11)
Etoposide	325.67	228.69	425.18	gamma	(11)
Carboplatin	65.87	32.61	123.90	gamma	(11)
Regular Radiation	26.33	19.75	32.91	gamma	(11)
Topotecan	77.36	58.02	96.70	gamma	(11)
Pembrolizumab	5026.09	3769.57	6282.61	gamma	(11)
Best supportive treatment	332.85	249.64	416.07	gamma	(10)
End of life	7985.90	5989.42	9982.37	gamma	(13)
Other cost per cycle ($)					
Laboratory testing	399.54	299.66	499.43	gamma	(16)
Imaging examination	168.07	126.05	210.09	gamma	(16)
Cost of managing adverse events ($)					
Neutropenia	85.37	64.02	106.71	gamma	(10)
Leukopenia	472.40	354.30	590.50	gamma	(10)
Thrombocytopenia	1068.48	801.36	1335.60	gamma	(10)
Anemia	515.18	386.38	643.97	gamma	(10)
Hypertriglyceridemia	122.29	91.72	152.86	gamma	(11)
Hypertension	176.34	132.26	220.43	gamma	(14)
Health utility and disutility					
Progression-free survival	0.8400	0.6300	0.9000	beta	(18)
Progressive disease	0.4730	0.3548	0.5913	beta	(19)
Neutropenia	-0.0910	-0.1138	-0.0683	beta	(19)
Leukopenia	-0.0910	-0.1138	-0.0683	beta	(19)
Thrombocytopenia	-0.1900	-0.2375	-0.1425	beta	(20)
Anemia	-0.0730	-0.0913	-0.0548	beta	(20)
Hypertriglyceridemia	-0.0270	-0.0338	-0.0203	beta	(14)
Hypertension	-0.0294	-0.1051	-0.0500	beta	(15)
Rate of increase in utility value of each group					
Benmelstobart+anlotinib+EC	0.0471	0.0353	0.0588	beta	(6)
Anlotinib+EC	0.0341	0.0256	0.0426	beta	(6)
EC	0.0190	0.0142	0.0237	beta	(6)
Risk of AEs in benmelstobart+anlotinib+EC group					
Neutropenia	0.6950	0.5213	0.8688	beta	(6)
Leukopenia	0.3820	0.2865	0.4775	beta	(6)
Thrombocytopenia	0.4960	0.3720	0.6200	beta	(6)
Anemia	0.2400	0.1800	0.3000	beta	(6)
Hypertension	0.1550	0.1163	0.1938	beta	(6)
Risk of AEs in anlotinib+EC group					
Neutropenia	0.7300	0.5475	0.9125	beta	(6)
Leukopenia	0.3070	0.2303	0.3838	beta	(6)
Thrombocytopenia	0.5370	0.4028	0.6713	beta	(6)
Anemia	0.2660	0.1995	0.3325	beta	(6)
Hypertriglyceridemia	0.0820	0.0615	0.1025	beta	(6)
Hypertension	0.1190	0.0893	0.1488	beta	(6)
Risk of AEs in EC group					
Neutropenia	0.6870	0.5153	0.8588	beta	(6)
Leukopenia	0.3460	0.2595	0.4325	beta	(6)
Thrombocytopenia	0.3580	0.2685	0.4475	beta	(6)
Anemia	0.2360	0.1770	0.2950	beta	(6)
Proportion of subsequent treatments in benmelstobart+anlotinib+EC group					
Radiotherapy	0.0690	0.0517	0.0862	beta	(6)
Chemotherapy	0.2091	0.1568	0.2614	beta	(6)
Targeted therapy	0.0798	0.0598	0.0997	beta	(6)
Immunotherapy	0.0690	0.0517	0.0862	beta	(6)
Best supportive treatment	0.5732	0.4299	0.7165	beta	(6)
Proportion of subsequent treatments in anlotinib+EC group					
Radiotherapy	0.0980	0.0735	0.1225	beta	(6)
Chemotherapy	0.2919	0.2189	0.3648	beta	(6)
Targeted therapy	0.1084	0.0813	0.1355	beta	(6)
Immunotherapy	0.0855	0.0641	0.1068	beta	(6)
Best supportive treatment	0.4163	0.3122	0.5204	beta	(6)
Proportion of subsequent treatments in EC group					
Radiotherapy	0.1131	0.0849	0.1414	beta	(6)
Chemotherapy	0.3494	0.2620	0.4367	beta	(6)
Targeted therapy	0.1449	0.1087	0.1811	beta	(6)
Immunotherapy	0.1052	0.0789	0.1315	beta	(6)
Best supportive treatment	0.2874	0.2156	0.3593	beta	(6)

AEs, adverse events; EC, etoposide plus carboplatin.

### 2.4 Health utility estimation

Due to the absence of explicit health utility values in the ETER701 trial, the utility value for PFS in extensive-stage small cell lung cancer was derived from a related study estimating utility values across all lung cancer subtypes (18). However, there are currently no utility values available for PD in extensive-stage small cell lung cancer; thus, the PD utility value was obtained from a health utility study on non-small cell lung cancer (19). The ETER701 trial indicated no significant differences in the changes in EuroQol visual analog scale scores among the three treatment groups relative to baseline, and the average change in scores over time generally increased (indicating improvement). Therefore, we assumed that the utility values for each state in the three treatment groups were consistent and adjusted the utility values based on score changes to reflect improvements in health status after treatment, while the utility values after the trial cutoff point remained unadjusted (6). The utility value loss associated with adverse events was derived from two cost-effectiveness analysis studies and two health utility studies on lung cancer (14, 15, 19, 20). Similar to costs, the utility value losses for adverse events were also adjusted based on their incidence rates. Relevant parameters are detailed in **Table 1**.

### 2.5 Basic analyses

Cost and quality-adjusted life years (QALYs) were used to measure the health benefits of the treatment interventions. According to pharmacoeconomic guidelines, both costs and QALYs were discounted at an annual rate of 5% in the base case analysis (21). Cost-effectiveness of the three treatment regimens was assessed by comparing the incremental cost-effectiveness ratio (ICER) against China’s willingness-to-pay (WTP) threshold. The WTP threshold was set at three times the per capita gross domestic product (GDP) of China (USD 37,598/QALY), in accordance with pharmacoeconomic guidelines (21).

### 2.6 Sensitivity and scenario analyses

One-way and probabilistic sensitivity analyses (PSA) were conducted to evaluate the robustness of the base case results and to explore the direction of influence of various factors. The estimated ranges for each parameter in the sensitivity analysis were based on confidence intervals, ranges, and standard deviations from the literature, or were set as baseline values ±25%. In the PSA, cost parameters were modeled using a gamma distribution, while probability and utility value parameters were modeled using a beta distribution. Correlations between certain parameters were also assumed, followed by a Monte Carlo simulation with 10,000 iterations (22). Specifically, within a correlation range of (-1, 1) (where 0 indicates no correlation, and values greater or less than 0 indicate positive or negative correlations, respectively), the correlation for utility values between groups based on score increases were set at 0.9, while the correlation between the proportions of chemotherapy and best supportive care in subsequent treatments was set at -0.9.

To explore the impact of alternative model settings on the analysis results, we also conducted scenario analyses. We performed a scenario analysis on the price of benmelstobart to determine the price reduction required for the benmelstobart plus anlotinib plus EC regimen to become cost-effective. Given that the overall survival (OS) hazard ratio (HR) between the anlotinib plus EC group and the EC group was not significant (HR = 0.86, 95% confidence interval: 0.67–1.10), we further conducted a scenario analysis on this HR value. This analysis primarily involved generating the OS curve for the anlotinib plus EC group by adjusting the OS curve of the EC group using the HR, allowing us to explore the specific impact of this HR variation on the cost-effectiveness analysis results and whether changes within the HR range would reverse the outcome. Additionally, we conducted scenario analyses for parameters such as the discount rate, study time horizon, adverse events, supportive care, palliative care, and the frequency of adverse events, making reasonable or conservative assumptions about the ranges and existence of these parameters.

## 3 Results

### 3.1 Basic analysis results

The base case analysis results indicated that at a WTP threshold of 37,598 USD/QALY, the regimen of benmelstobart plus anlotinib plus EC was not cost-effective compared to either anlotinib plus EC or EC alone, whereas anlotinib plus EC was cost-effective compared to EC. In terms of cost breakdown, the total cost for the benmelstobart plus anlotinib plus EC group was primarily driven by the costs of treatment drugs during the PFS period, while the total costs for the anlotinib plus EC and EC groups were mainly accumulated from treatment drug costs during the PD period. Detailed results of the base case analysis are presented in **Table 2**.

**Table 2:** Basic analysis results.

Parameter	Benmelstobart+anlotinib+EC	Anlotinib+EC	EC
Total life years	2.0843	1.4631	1.2176
QALYs	1.4662	0.9325	0.7374
Total cost ($)	103,259.96	26,488.69	22,380.83
Incremental cost ($)			
vs.EC	80,879.12	4,107.86	–
vs. anlotinib+EC	76,771.27	–	–
Incremental QALYs			
vs. EC	0.7288	0.1951	–
vs. anlotinib+EC	0.5337	–	–
ICER			
vs. EC	110,970.19	21,056.19	–
vs. anlotinib+EC	143,834.77	–	–
Cost of PFS drugs	82,502.10	5,764.58	1,516.66
Cost of PD drugs	8,144.89	8,483.47	9,127.29
Cost of Adverse Event Management	919.91	948.80	723.75
Cost of Follow up	4,697.52	3,938.77	3,532.26
Cost of end-of-life	6,995.54	7,353.06	7,480.88

EC, etoposide plus carboplatin; QALYs, quality-adjusted life years; ICER, incremental cost-effectiveness ratio; PFS, progression-free survival; PD, progressive disease.

### 3.2 Sensitivity analyses results

**Figure 2:**
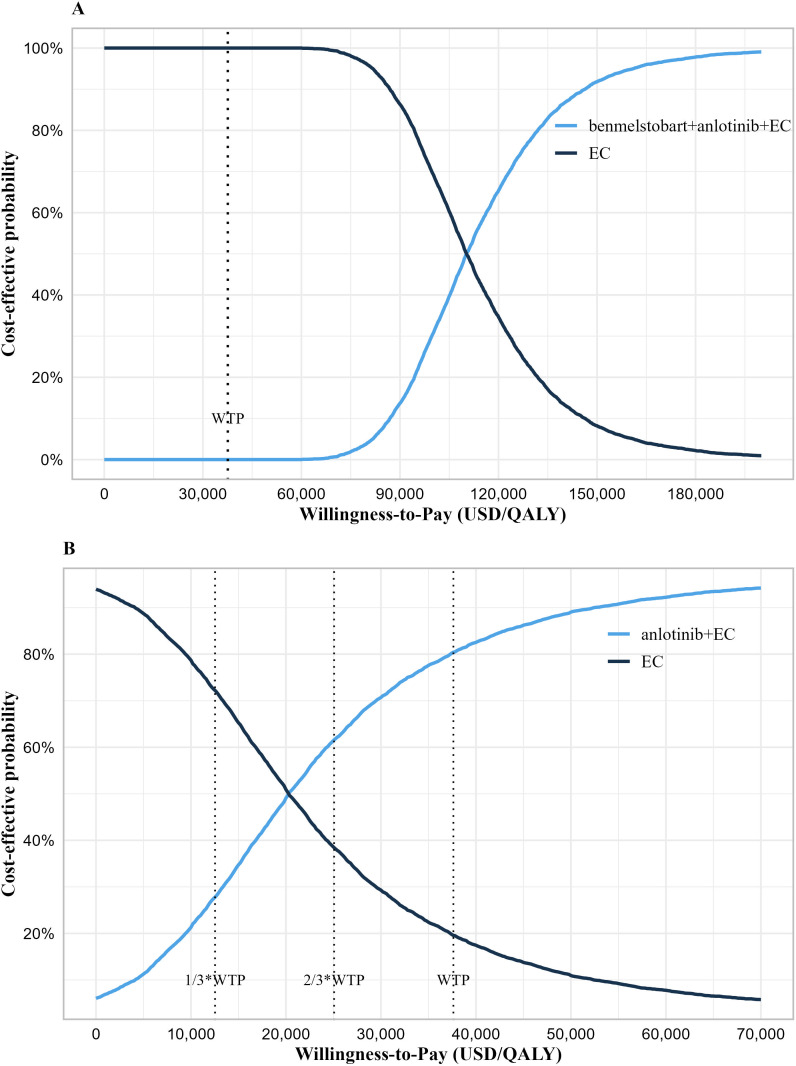
Cost-effectiveness acceptability curve. **(A)** Cost-effectiveness acceptability curve of the benmelstobart plus anlotinib plus EC group vs. EC group. **(B)** Cost-effectiveness acceptability curve of the anlotinib plus EC group vs. EC group. WTP, willingness-to-pay; QALY, quality-adjusted life years; EC, etoposide plus carboplatin.

The results of probability sensitivity analysis are shown in **Figure 2** and **Supplementary Figure 2**. Under the condition of random sampling of all parameters, the results of the probabilistic sensitivity analysis indicated that at a WTP threshold of 37,598 USD/QALY, the probability of benmelstobart plus anlotinib plus EC being cost-effective was 0%, while the probability for anlotinib plus EC was as high as 80.42%. When the WTP thresholds were set at 25,065 USD/QALY and 12,533 USD/QALY, the probabilities of anlotinib plus EC being cost-effective were 61.64% and 27.74%, respectively. The analysis results were robust at a WTP threshold of 37,598 USD/QALY.

**Figure 3:**
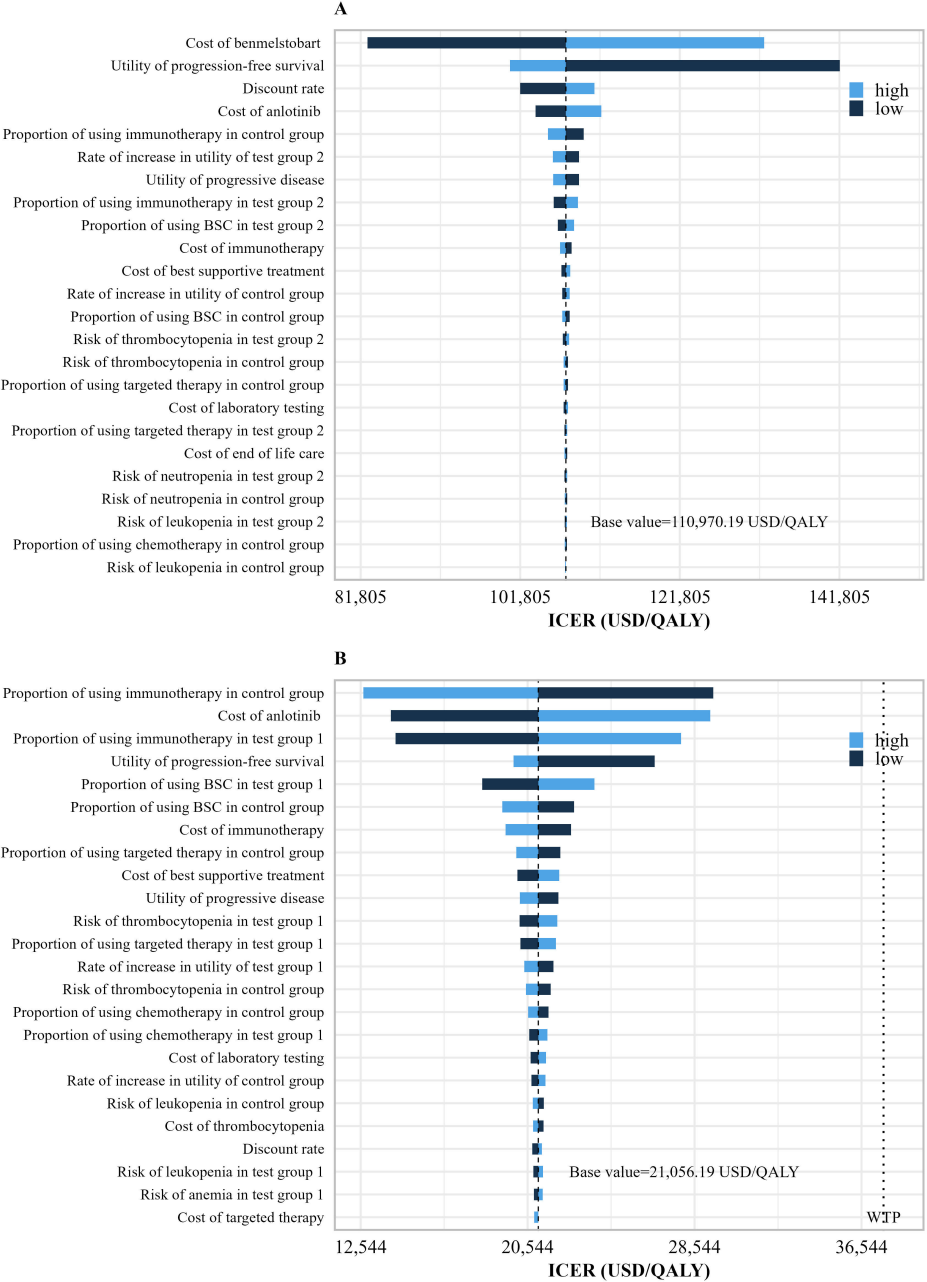
Univariate sensitivity analysis results. **(A)** Univariate sensitivity analysis results of the benmelstobart plus anlotinib plus EC group vs. EC group. **(B)** Univariate sensitivity analysis results of the anlotinib plus EC group vs. EC group. WTP, willingness-to-pay; QALY, quality-adjusted life years; Tset group 2, benmelstobart+anlotinib+EC; Tset group 1, anlotinib+EC; Tset group 1, EC alone.

The results of univariate sensitivity analysis are shown in **Figure 3**. The one-way sensitivity analysis displayed only the parameters that had a significant impact on the results. It showed that for the benmelstobart plus anlotinib plus EC group, the parameters that most significantly influenced the ICER were the PFS utility value and the price of benmelstobart. In the anlotinib plus EC group, the parameters with considerable influence included the cost and proportion of immunotherapy, the price of anlotinib, the proportion of best supportive care, and the PFS utility value.

### 3.3 Scenario analyses results

The results of the scenario analysis indicated that a price reduction of 73.79% for benmelstobart would render the regimen of benmelstobart plus anlotinib plus EC potentially cost-effective. Adjusting the OS curve of the EC group using the HR to generate the OS curve for the anlotinib plus EC group had minimal impact on the base case results. Specifically, when the HR decreased, the ICER increased, and when the HR increased, the ICER decreased. When the study time horizon was set to match the duration of the ETER701 trial (2 years), the ICER for the anlotinib plus EC group increased, while the ICER for the benmelstobart plus anlotinib plus EC group decreased. The scenario analysis that excluded best supportive care indicated a reduction in the ICER for both the benmelstobart plus anlotinib plus EC group and the anlotinib plus EC group. Since best supportive care was not included in the subsequent treatment of the ETER701 trial, this scenario may better align with the survival benefits observed in the three treatment groups. Additional results from the scenario analysis are detailed in **Table 3**.

**Table 3:** Scenario analysis results.

Scene	Total cost	QALYs	ICER	ICER changes
Base case				
benmelstobart+anlotinib+EC	103,259.96	1.4662	110,970.19	–
anlotinib+EC	26,488.69	0.9325	21,056.19	–
EC	22,380.83	0.7374	–	–
Benmelstobart price ($3444.6)				
2066.76$ (price*0.6)	74,270.43	1.4662	71,195.11	↓
922.12$ (price*0.2621)	49,781.52	1.4662	37,595.12	↓(<WTP)
Changes in OS curve of anlotinib+EC group (generated by adjusting the OS curve of EC group with HR)				
HR: 0.67	28,367.57	1.0489	19,219.36	↓
HR: 0.86	25,871.70	0.9084	20,406.94	↓
HR: 1.10	23,817.45	0.8120	19,260.30	↓
Discount rate				
Only utility values were discounted				
benmelstobart+anlotinib+EC	113,144.86	1.4662	122,944.82	↑
anlotinib+EC	28,011.19	0.9325	22,927.85	↑
EC	23,538.19	0.7374	–	
Only costs were discounted				
benmelstobart+anlotinib+EC	103,259.96	1.6227	94,980.71	↓
anlotinib+EC	26,488.69	0.9865	19,076.90	↓
EC	22,380.83	0.7712	–	
Not discounted at all				
benmelstobart+anlotinib+EC	113,144.86	1.6227	105,229.94	↓
anlotinib+EC	28,011.19	0.9865	20,772.62	↓
EC	23,538.19	0.7712	–	
Research deadline				
5 years				
benmelstobart+anlotinib+EC	94,352.11	1.3234	120,722.47	↑
anlotinib+EC	26,136.95	0.9096	21,415.22	↑
EC	22,198.92	0.7257	–	
2 years				
benmelstobart+anlotinib+EC	72,262.37	0.9691	170,310.44	↑
anlotinib+EC	22,522.47	0.7884	18,033.82	↓
EC	20,276.47	0.6638	–	
No best supportive care				
benmelstobart+anlotinib+EC	100,252.60	1.4662	108,770.80	↓
anlotinib+EC	24,303.60	0.9325	17,054.31	↓
EC	20,976.47	0.7374	–	
No end-of-life care				
benmelstobart+anlotinib+EC	96,264.42	1.4662	111,636.11	↑
anlotinib+EC	19,135.63	0.9325	21,711.38	↑
EC	14,899.95	0.7374	–	
Utility values were not adjusted				
benmelstobart+anlotinib+EC	103,232.65	1.4235	115,760.82	↑
anlotinib+EC	26,457.69	0.9070	22,407.24	↑
EC	22,380.83	0.7251	–	
AEs occurred once again at the beginning of the PD phase				
benmelstobart+anlotinib+EC	104,105.88	1.4662	111,158.65	↑
anlotinib+EC	27,404.84	0.9325	22,120.22	↑
EC	23,089.40	0.7374	–	

EC, etoposide plus carboplatin; QALYs, quality-adjusted life years; ICER, incremental cost-effectiveness ratio; AEs, adverse events; PD, progressive disease. WTP, willingness-to-pay. ↑, Indicates that the ICER in this scenario is higher than the ICER in the base case; ↓, Indicates that the ICER in this scenario is lower than the ICER in the base case.

## 4 Discussion

To our knowledge, this study is the first to evaluate the cost-effectiveness of the benmelstobart plus anlotinib combined with etoposide and carboplatin regimen compared to the anlotinib combined with etoposide and carboplatin regimen, as well as etoposide and carboplatin alone. The benmelstobart plus anlotinib plus EC treatment regimen in the ETER701 trial set a new record for overall survival in patients with ES-SCLC. However, based on the analysis in this study, the high cost of benmelstobart renders the clinical benefits of this regimen not cost-effective. In contrast, the anlotinib plus EC treatment regimen provides an additional 0.1951 QALYs at a cost of only $4,107.86, resulting in an ICER of 21,056.19 USD/QALY, with a cost-effectiveness probability of up to 80.42% under the WTP threshold of 37,598 USD/QALY.

Both the one-way sensitivity analysis and probability sensitivity analysis results indicate that the evaluation outcomes in this study are robust under the WTP threshold. The results of the one-way sensitivity analysis can be validated through cost breakdown. In the benmelstobart plus anlotinib plus EC group, the cost of benmelstobart constitutes a significant proportion of the total cost, which gives its price substantial influence and renders the ICER insensitive to variations in other parameters. Additionally, as a recurring cost, the cost of benmelstobart also means that the discount rate has a certain impact on the ICER. In the comparison between anlotinib plus EC and EC, the PD costs account for a substantial proportion of the total costs in both groups, with the cost of immunotherapy being particularly significant within the PD costs. Therefore, the cost and proportion of immunotherapy have the greatest impact on the ICER. Although the cost of anlotinib accounts for only 20% of the anlotinib plus EC group, its nature as a recurring cost means that fluctuations in its value also have a considerable effect on the ICER. Compared to the EC group, both the benmelstobart plus anlotinib plus EC and anlotinib plus EC groups demonstrate significant clinical benefits during the PFS period, making the PFS utility value a major influencing factor in both analyses.

In the scenario analysis, a 73.79% reduction in the price of benmelstobart to $922.12 per 1200 mg would make the benmelstobart plus anlotinib plus EC regimen potentially cost-effective. Although the introduction of anlotinib aims to enhance the efficacy of benmelstobart, leading to increased costs compared to the first-line recommended treatment for ES-SCLC, atezolizumab plus EC, the cycle cost of the benmelstobart and anlotinib combination remains lower than that of atezolizumab in China (approximately $3,848 for benmelstobart plus anlotinib compared to approximately $4,600 for atezolizumab) (4, 12). Furthermore, the benmelstobart plus anlotinib plus EC regimen has made significant progress in improving overall survival for ES-SCLC patients, setting new records. A network meta-analysis and cost-effectiveness analysis from a U.S. perspective regarding first-line treatment for ES-SCLC indicate that atezolizumab plus chemotherapy provides the highest incremental QALYs (0.25 QALYs) compared to chemotherapy alone, which is considerably lower than the incremental QALYs of 0.7288 observed for the benmelstobart plus anlotinib plus EC regimen in this study (23). The PFS utility value of 0.673 used in the U.S. cost-effectiveness analysis comes from a health status study of non-small cell lung cancer (NSCLC) in the UK (19). If the PFS utility value from this study is replaced with 0.673, the incremental QALYs for the benmelstobart plus anlotinib plus EC regimen would still be 0.58. Additionally, a cost-effectiveness analysis from a Chinese perspective of atezolizumab combined with chemotherapy for first-line treatment of NSCLC reported an ICER of 267,264.85 USD/QALY, which is significantly higher than the ICER for the benmelstobart plus anlotinib plus EC regimen in this study (24). Based on these factors, the benmelstobart plus anlotinib plus EC regimen may demonstrate superior cost-effectiveness compared to the atezolizumab plus EC regimen.

Anlotinib plus EC has shown potential cost-effectiveness compared to EC alone, but the certainty of its clinical benefits remains questionable, primarily due to the HR confidence interval encompassing 1. In the sensitivity analysis of HR, we observed that setting the HR value to its baseline level (0.86) resulted in a decrease in both total costs and QALYs for the anlotinib plus EC group. This discrepancy may stem from the underestimation of clinical benefits during the later stages of follow-up when using the OS curve generated by the HR parameter compared to the KM curve observed in clinical trials. Conversely, distribution models can more accurately fit the KM curves from clinical trials, thus providing a more reliable representation of clinical benefits. In further analysis, we observed an interesting phenomenon: when the clinical benefit of snlotinib plus EC in terms of OS was lower than that of EC (with an HR of 1.10), the ICER for the snlotinib plus EC group significantly decreased. We speculate that this is related to the fact that PD costs account for the largest proportion of total costs in both the snlotinib plus EC and EC groups. Specifically, as the HR increases, the OS curve shifts downward, resulting in a reduced duration or number of patients in the PD state, thereby lowering PD costs. Although QALYs during the PD period also decrease accordingly, the reduction in costs outweighs the reduction in QALYs due to the lower utility value of the PD state, leading to a significant drop in ICER.

In the current study, the total cost during the PD phase is primarily constituted by subsequent treatments. Although not all PD patients received further treatment in the ETER701 trial, this study assumes that patients not receiving subsequent treatment received the best supportive care. This assumption may lead to an overestimation of the total cost during the PD phase. In reality, the costs for PD patients who did not receive best supportive care might more closely reflect the clinical benefits observed in the three treatment groups. In scenarios without best supportive care, the ICERs are found to decrease. This is because, in the ETER701 trial, 71% of PD patients in the EC group underwent subsequent treatment, which is higher compared to the other two groups (43% and 58%). Without best supportive care, the cost advantage for the benmelstobart plus anlotinib plus EC and anlotinib plus EC groups increases. Therefore, based on the scenario analysis results for HR and best supportive care, the cost-effectiveness of anlotinib plus EC remains robust.

In this study, we incorporated flexible parametric models to fit the KM curves. Compared to some standard parametric models, most flexible parametric models demonstrate better goodness of fit (25). Therefore, flexible parametric models can better evaluate the clinical benefits of each treatment regimen. We further considered the changes in EuroQol Visual Analogue Scale scores from baseline in the ETER701 trial and adjusted health utility values accordingly using these change rates. By accounting for the change rates in health utility values, we aimed to more accurately reflect the improvement in health-related quality of life following treatment interventions. In the scenario analysis without adjusting utility values, we observed a slight increase in the ICER, but this increase was not sufficient to reverse the cost-effectiveness evaluation results.

The model analysis in this study has inherent limitations that need to be acknowledged. First, the model’s predictions largely rely on a phase III clinical trial (the ETER701 trial). However, this trial did not provide IPD, limiting our ability to conduct direct data analysis. To address this limitation, we used the IPDfromKM package to reconstruct pseudo-IPD and employed parametric models to fit and extrapolate KM curves for predicting long-term outcomes (26). Although the maturity of KM curves in the ETER701 trial is generally high (greater than 60%), with the PFS KM curve exceeding 80%, there is still uncertainty in long-term outcome predictions. Secondly, the ETER701 trial did not provide detailed information on post-progression treatment regimens and adverse events. This includes specifics on the subsequent treatment regimens, drug choices, treatment durations, and the frequency and duration of adverse events. In this study, we could only make reasonable assumptions based on available data and clinical experience, which may differ from actual conditions, leading to slight discrepancies in costs and QALYs. Lastly, given the significant disparities in development levels across various regions in China, which lead to varying willingness-to-pay (WTP) thresholds, the evaluation results of this study have certain limitations. Nevertheless, decision-makers in each region can utilize the outcomes of the probability sensitivity analysis to assess the cost-effectiveness of strategies at different WTP threshold levels.

## 5 Conclusion

Through model analysis, the ICER of benmelstobart plus anlotinib plus EC and anlotinib plus EC compared to EC alone was $110,970.19 per QALY and $21,056.19 per QALY, respectively. Compared to anlotinib plus EC, the ICER for benmelstobart plus anlotinib plus EC was $143,834.77 per QALY. Based on the WTP threshold of $37,598 per QALY in China, benmelstobart plus anlotinib plus EC is not a cost-effective first-line treatment option for Chinese patients with ES-SCLC, whereas anlotinib plus EC is cost-effective.

## Data Availability

The original contributions presented in the study are included in the article/**Supplementary Material**. Further inquiries can be directed to the corresponding authors.
