# Supplementary material for: Cost-effectiveness analysis of anlotinib plus chemotherapy with or without benmelstobart versus chemotherapy alone for extensive-stage small-cell lung cancer in China
Source: Front Oncol. 2024 Dec 17;14:1484650. doi: 10.3389/fonc.2024.1484650 (PMC11685082; doi:10.3389/fonc.2024.1484650)
Supplement: Supplementary file 1 [file DataSheet1.docx]

Supplementary Material

# eTable 1. CHEERS 2022 Checklist

# eTable 2. Survival curve fitting information and goodness of fit

# eFigure 1. KM curve reconstruction and parameter model extrapolation diagram

# eFigure 2. Probability sensitivity analysis results. (A) The probability sensitivity analysis results of the benmelstobart plus anlotinib plus EC group vs. EC group. (B) The probability sensitivity analysis results of the anlotinib plus EC group vs. EC group. QALYs, quality-adjusted life years; GDP, per capita gross domestic product.

# eMethod 1 Time-varying Markov models R code

# Supplementary Figures and Tables

## Supplementary Table

**eTable 1.** CHEERS 2022 Checklist

|  | **Item** | **Guidance for Reporting** | **Is it reported?** |
| --- | --- | --- | --- |
| **Title** | | |  |
| Title | 1 | Identify the study as an economic evaluation and specify the interventions being compared. | √ |
| **Abstract** | | |  |
| Abstract | 2 | Provide a structured summary that highlights context, key methods, results, and alternative analyses. | √ |
| **Introduction** | | |  |
| Introduction: Background and Objectives | 3 | Give the context for the study, the study question, and its practical relevance for decision making in policy or practice. | √ |
| **Methods** | | |  |
| Health economic analysis plan | 4 | Indicate whether a health economic analysis plan was developed and where available. | √ |
| Study population | 5 | Describe characteristics of the study population (such as age range, demographics, socioeconomic, or clinical characteristics). | √ |
| Setting and location | 6 | Provide relevant contextual information that may influence findings. | √ |
| Comparators | 7 | Describe the interventions or strategies being compared and why chosen. | √ |
| Perspective | 8 | State the perspective(s) adopted by the study and why chosen. | √ |
| Time horizon | 9 | State the time horizon for the study and why appropriate. | √ |
| Discount rate | 10 | Report the discount rate(s) and reason chosen. | √ |
| Selection of outcomes | 11 | Describe what outcomes were used as the measure(s) of benefit(s) and harm(s). | √ |
| Measurement of outcomes | 12 | Describe how outcomes used to capture benefit(s) and harm(s) were measured. | √ |
| Valuation of outcomes | 13 | Describe the population and methods used to measure and value outcomes. | √ |
| Measurement and valuation of resources and costs | 14 | Describe how costs were valued. | √ |
| Currency, price date, and conversion | 15 | Report the dates of the estimated resource quantities and unit costs, plus the currency and year of conversion. | √ |
| Rationale and description of model | 16 | If modeling is used, describe in detail and why used. Report if the model is publicly available and where it can be accessed. | √ |
| Analytics and assumptions | 17 | Describe any methods for analyzing or statistically transforming data, any extrapolation methods, and approaches for validating any model used. | √ |
| Characterizing heterogeneity | 18 | Describe any methods used for estimating how the results of the study vary for subgroups. | NA |
| Characterizing distributional effects | 19 | Describe any methods used for estimating how the results of the study vary for subgroups. | NA |
| Characterizing uncertainty | 20 | Describe methods to characterize any sources of uncertainty in the analysis. | √ |
| Approach to engagement with patients and others affected by the study | 21 | Describe any approaches to engage patients or service recipients, the general public, communities, or stakeholders (eg, clinicians or payers) in the design of the study. | × |
| **Results** | | |  |
| Study parameters | 22 | Report all analytic inputs (eg, values, ranges, references) including uncertainty or distributional assumptions. | √ |
| Summary of main results | 23 | Report the mean values for the main categories of costs and outcomes of interest and summarize them in the most appropriate overall measure. | √ |
| Effect of uncertainty | 24 | Describe how uncertainty about analytic judgments, inputs, or projections affects findings. Report the effect of choice of discount rate and time horizon, if applicable. | √ |
| Effect of engagement with patients and others affected by the study | 25 | Report on any difference patient/service recipient, general public, community, or stakeholder involvement made to the approach or findings of the study. | NA |
| **Discussion** | | |  |
| Study findings, limitations, generalizability, and current knowledge | 26 | Report key findings, limitations, ethical, or equity considerations not captured and how these could impact patients, policy, or practice. | √ |
| **Other Relevant Information** | | |  |
| Source of funding | 27 | Describe how the study was funded and any role of the funder in the identification, design, conduct, and reporting of the analysis. | √ |
| Conflicts of interest | 28 | Report authors’ conflicts of interest according to journal or International Committee of Medical Journal Editors requirements. | √ |

**eTable 2.** Survival curve fitting information and goodness of fit

| Group | Model | AIC | BIC | Additional information |
| --- | --- | --- | --- | --- |
| OS | | | | |
| Benmelstobart+Anlotinib+EC | RP-normal | 117.30 | 124.31 | gamma0=-0.42; gamma1=1.03; knot= 0; scale= normal |
| Anlotinib+EC | RP-hazard | 122.41 | 136.41 | gamma0=-3.29; gamma1=0.66; gamma2=-1.57; gamma3=2.11; knot= 2; scale= hazard |
| EC | RP-odds | 135.40 | 145.93 | gamma0=-1.3; gamma1=1.49; gamma2=-0.17; knot= 1; scale= odds |
| PFS | | | | |
| Benmelstobart+Anlotinib+EC | RP-hazard | 118.15 | 142.69 | gamma0=-0.23; gamma1=2.13; gamma2=-44.95; gamma3=64.35; gamma4=-38.9; gamma5=26.08; gamma6=-6.59; knot= 5; scale= hazard |
| Anlotinib+EC | log-logistic | 118.54 | 125.55 | shape=1.14; scale=-0.66 |
| EC | RP-hazard | 120.68 | 134.72 | gamma0=-2.29; gamma1=0.94; gamma2=-18.21; gamma3=19.74; knot= 2; scale= hazard |

OS, overall survival; PFS, progression-free survival; EC, etoposide-carboplatin; RP-hazard, the 'hazard' Royston-Parmar model; RP-odds, the ' odds ' Royston-Parmar model; RP-normal, the 'normal' Royston-Parmar model; llogis, log-logistic.

##
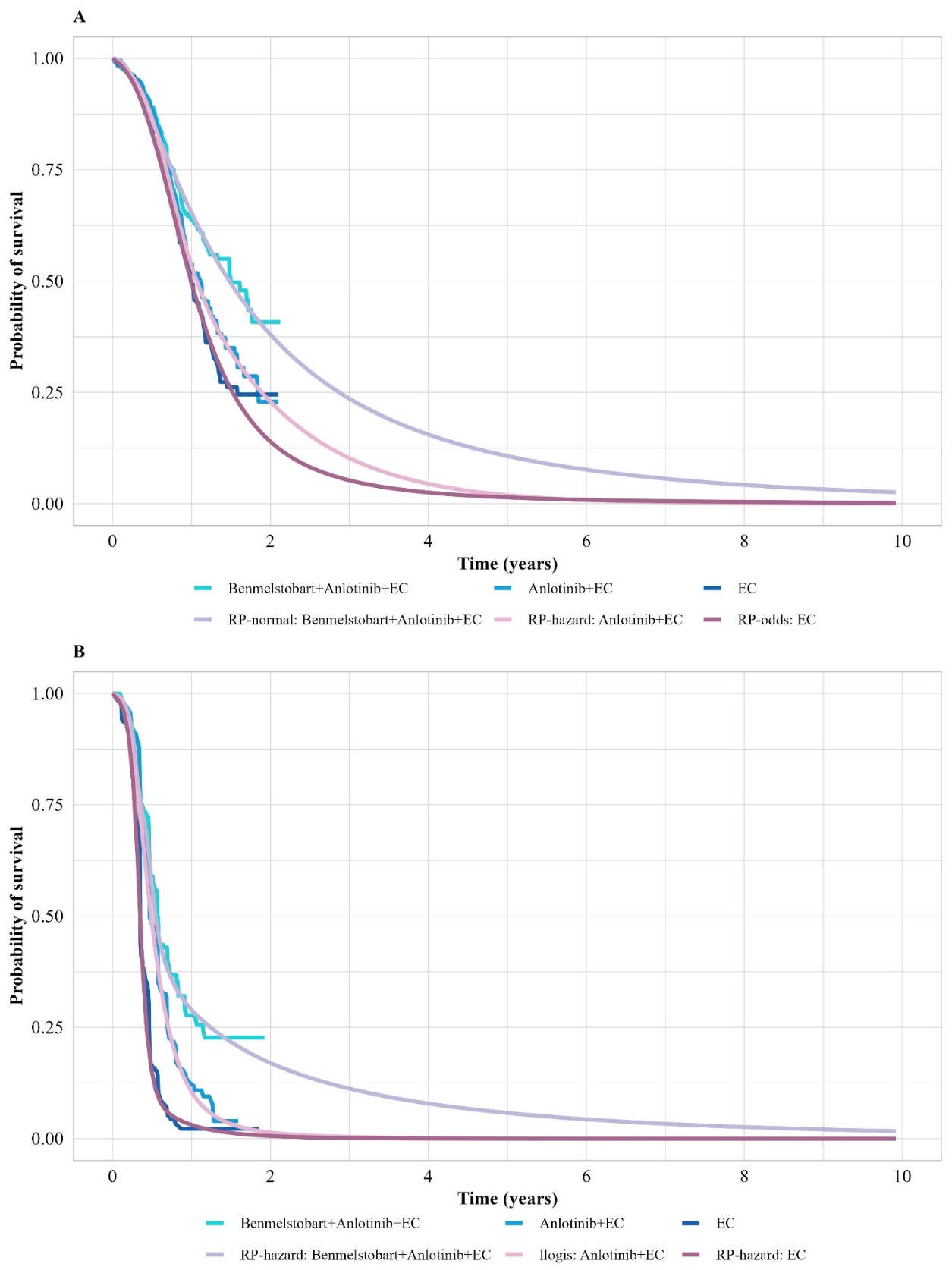
Supplementary Figures

**eFigure 1.** KM curve reconstruction and parameter model extrapolation diagram. (A) KM curve reconstruction and parameter model extrapolation diagram. (B) . KM, Kaplan-Meier; OS, overall survival; PFS, progression-free survival; EC, etoposide-carboplatin; RP-hazard, the 'hazard' Royston-Parmar model; RP-odds, the ' odds ' Royston-Parmar model; RP-normal, the 'normal' Royston-Parmar model; llogis, log-logistic.


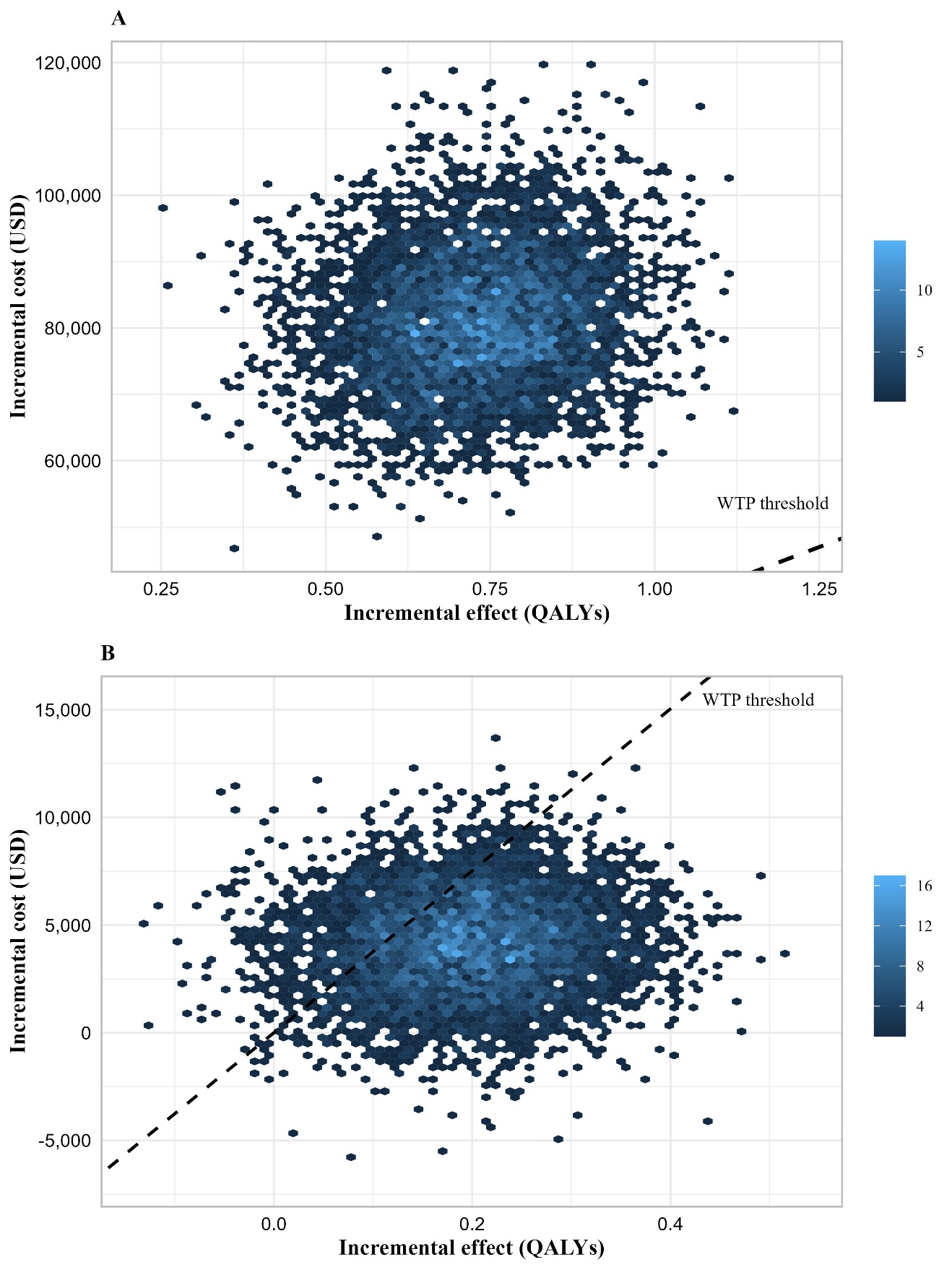


**eFigure 2.** Probability sensitivity analysis results. (A) The probability sensitivity analysis results of the benmelstobart plus anlotinib plus EC group vs. EC group. (B) The probability sensitivity analysis results of the anlotinib plus EC group vs. EC group. QALYs, quality-adjusted life years; WTP, willingness-to-pay.

**eMethod 1.** Time-varying Markov models R code

## Read transition probability and model parameter data

TG2 <- read.xlsx("Transition probabilities between states in benmelstobart+anlotinib+EC group.xlsx")

TG1 <- read.xlsx("Transition probabilities between states in anlotinib+EC group.xlsx")

CG <- read.xlsx("Transition probabilities between states in EC group.xlsx")

par1 <- read.xlsx("Model input parameters.xlsx")

## Define transition probability matrix

mat_TG2 <- define_transition(

state_names = c("pfs", "pd", "d"),

TG2[model_time,"pFTF"], TG2[model_time,"pFTP"], TG2[model_time,"pFTD"],

0, TG2[model_time,"pPTP"],TG2[model_time,"pPTD"],

0,0,1)

mat_TG1 <- define_transition(

state_names = c("pfs", "pd", "d"),

TG1[model_time,"pFTF"], TG1[model_time,"pFTP"], TG1[model_time,"pFTD"],

0, TG1[model_time,"pPTP"],TG1[model_time,"pPTD"],

0,0,1)

mat_CG <- define_transition(

state_names = c("pfs", "pd", "d"),

CG[model_time,"pFTF"], CG[model_time,"pFTP"], CG[model_time,"pFTD"],

0, CG[model_time,"pPTP"],CG[model_time,"pPTD"],

0,0,1)

##Define model state

state_pfs <- define_state(

cost_treat = discount(dispatch_strategy(

TG2 = C_ben+C_anl+C_eto+C_car,

TG1 = C_anl+C_eto+C_car,

CG = C_eto+C_car),r = dr/(365.24/21)),

cost_other = discount(C_test+C_image, r = dr/(365.24/21)),

cost_ae = discount(dispatch_strategy(

TG2 = C_neu*R_neu2+C_leu*R_leu2+C_thr*R_thr2+C_ane*R_ane2+C_hyp*R_hyp2+C_tri*R_tri2,

TG1 = C_neu*R_neu1+C_leu*R_leu1+C_thr*R_thr1+C_ane*R_ane1+C_hyp*R_hyp1+C_tri*R_tri1,

CG = C_neu*R_neu0+C_leu*R_leu0+C_thr*R_thr0+C_ane*R_ane0+C_hyp*R_hyp0+C_tri*R_tri0), r = dr/(365.24/21)),

cost_eol = 0,

cost_total = cost_treat + cost_other+cost_ae+cost_eol,

utility= discount(dispatch_strategy(

TG2=(U_pfs-(U_neu*R_neu2+U_leu*R_leu2+U_thr*R_thr2+U_ane*R_ane2+U_hyp*R_hyp2+U_tri*R_tri2))/(365.24/21)*R_utg2,

TG1=(U_pfs-(U_neu*R_neu1+U_leu*R_leu1+U_thr*R_thr1+U_ane*R_ane1+U_hyp*R_hyp1+U_tri*R_tri1))/(365.24/21)*R_utg1,

CG=(U_pfs-(U_neu*R_neu0+U_leu*R_leu0+U_thr*R_thr0+U_ane*R_ane0+U_hyp*R_hyp0+U_tri*R_tri0))/(365.24/21)*R_ucg),r = dr/(365.24/21))

)

state_pd <- define_state(

cost_treat = discount(dispatch_strategy(

TG2 = C_rad*R_rad2+C_che*R_che2+C_tar*R_tar2+C_imu*R_imu2+C_bsc*R_bsc2,

TG1 = C_rad*R_rad1+C_che*R_che1+C_tar*R_tar1+C_imu*R_imu1+C_bsc*R_bsc1,

CG = C_rad*R_rad0+C_che*R_che0+C_tar*R_tar0+C_imu*R_imu0+C_bsc*R_bsc0),r = dr/(365.24/21)),

cost_other = discount(C_test+C_image, r = dr/(365.24/21)),

cost_ae = 0,

cost_eol = 0,

cost_total = cost_treat + cost_other+cost_ae+cost_eol,

utility= discount(dispatch_strategy(

TG2=(U_pd)/(365.24/21)*R_utg2,

TG1=(U_pd)/(365.24/21)*R_utg1,

CG=(U_pd)/(365.24/21)*R_ucg),r = dr/(365.24/21)))

state_d <- define_state(

cost_treat = 0,

cost_other = 0,

cost_ae = 0,

cost_eol =discount(C_eol, r = dr/(365.24/21)),

cost_total = cost_treat + cost_other+cost_ae+cost_eol,

utility= 0)

##Define strategy

strat_TG2 <- define_strategy(

transition = mat_TG2,

pfs = state_pfs,

pd = state_pd,

d = state_d)

strat_TG1 <- define_strategy(

transition = mat_TG1,

pfs = state_pfs,

pd = state_pd,

d = state_d)

strat_CG <- define_strategy(

transition = mat_CG,

pfs = state_pfs,

pd = state_pd,

d = state_d)

## Define model input parameters and run the model

res_mod2<- run_model(

TG2 = strat_TG2, TG1=strat_TG1,CG = strat_CG,

parameters = define_parameters (

C_ben= par1$base[par1$par == "C_ben"],

C_anl= par1$base[par1$par == "C_anl"],

C_eto= ifelse(model_time<=4,par1$base[par1$par == "C_eto"],0),

C_car= ifelse(model_time<=4,par1$base[par1$par == "C_car"],0),

C_bsc= par1$base[par1$par == "C_bsc"],

R_bsc2= par1$base[par1$par == "R_bsc2"],

R_bsc1= par1$base[par1$par == "R_bsc1"],

R_bsc0= par1$base[par1$par == "R_bsc0"],

C_rad= par1$base[par1$par == "C_rad"],

R_rad2= par1$base[par1$par == "R_rad2"],

R_rad1= par1$base[par1$par == "R_rad1"],

R_rad0= par1$base[par1$par == "R_rad0"],

C_che= par1$base[par1$par == "C_che"],

R_che2= par1$base[par1$par == "R_che2"],

R_che1= par1$base[par1$par == "R_che1"],

R_che0= par1$base[par1$par == "R_che0"],

C_tar= par1$base[par1$par == "C_tar"],

R_tar2= par1$base[par1$par == "R_tar2"],

R_tar1= par1$base[par1$par == "R_tar1"],

R_tar0= par1$base[par1$par == "R_tar0"],

C_imu= ifelse(state_time<35,par1$base[par1$par == "C_imu"],C_bsc),

R_imu2= par1$base[par1$par == "R_imu2"],

R_imu1= par1$base[par1$par == "R_imu1"],

R_imu0= par1$base[par1$par == "R_imu0"],

C_test= ifelse(model_time<365.24/21,par1$base[par1$par == "C_test"]*0.7/2,

ifelse(model_time<365.24*2/21,par1$base[par1$par == "C_test"]*0.7/3,

ifelse(model_time<365.24*4/21,par1$base[par1$par == "C_test"]*0.7/6,par1$base[par1$par == "C_test"]*0.7/12))),

C_image= ifelse(model_time<365.24/21,par1$base[par1$par == "C_image"]*0.7/2,

ifelse(model_time<365.24*2/21,par1$base[par1$par == "C_image"]*0.7/3,

ifelse(model_time<365.24*4/21,par1$base[par1$par == "C_image"]*0.7/6,par1$base[par1$par == "C_image"]*0.7/12))),

C_eol= ifelse(state_time<=1,par1$base[par1$par == "C_eol"],0),

C_neu= ifelse(model_time<=1,par1$base[par1$par == "C_neu"],0),

C_leu= ifelse(model_time<=1,par1$base[par1$par == "C_leu"],0),

C_thr= ifelse(model_time<=1,par1$base[par1$par == "C_thr"],0),

C_ane= ifelse(model_time<=1,par1$base[par1$par == "C_ane"],0),

C_hyp= ifelse(model_time<=1, C_hyp= par1$base[par1$par == "C_hyp"],0),

C_tri= ifelse(model_time<=1, C_tri= par1$base[par1$par == "C_tri"],0),

R_neu2= par1$base[par1$par == "R_neu2"],

R_neu1= par1$base[par1$par == "R_neu1"],

R_neu0= par1$base[par1$par == "R_neu0"],

R_leu2= par1$base[par1$par == "R_leu2"],

R_leu1= par1$base[par1$par == "R_leu1"],

R_leu0= par1$base[par1$par == "R_leu0"],

R_thr2= par1$base[par1$par == "R_thr2"],

R_thr1= par1$base[par1$par == "R_thr1"],

R_thr0= par1$base[par1$par == "R_thr0"],

R_ane2= par1$base[par1$par == "R_ane2"],

R_ane1= par1$base[par1$par == "R_ane1"],

R_ane0= par1$base[par1$par == "R_ane0"],

U_neu= ifelse(model_time<=1,par1$base[par1$par == "U_neu"],0),

U_leu= ifelse(model_time<=1,par1$base[par1$par == "U_leu"],0),

U_thr= ifelse(model_time<=1,par1$base[par1$par == "U_thr"],0),

U_ane= ifelse(model_time<=1,par1$base[par1$par == "U_ane"],0),

U_hyp= ifelse(model_time<=1, par1$base[par1$par == "U_hyp"],0),

U_tri= ifelse(model_time<=1, par1$base[par1$par == "U_tri"],0),

U_pfs= par1$base[par1$par == "U_pfs"],

U_pd= par1$base[par1$par == "U_pd"],

R_utg2=ifelse(model_time<=34,par1$base[par1$par == "R_utg2"]+1,1),

R_utg1=ifelse(model_time<=34,par1$base[par1$par == "R_utg1"]+1,1),

R_ucg=ifelse(model_time<=34,par1$base[par1$par == "R_ucg"]+1,1),

dr=par1$base[par1$par == "dr"] ),

cycles = 173,

cost = cost_total, effect = utility,

state_time_limit = c(pd = 34, d=1),

method = "life-table",

init = c(1000,0,0))
